# Identification of 2,4-Di-*tert*-butylphenol as a Novel Agonist for Insect Odorant Receptors

**DOI:** 10.3390/ijms25010220

**Published:** 2023-12-22

**Authors:** Shinhui Lee, Sanung Eom, Minsu Pyeon, Myungmi Moon, Jihwon Yun, Jaehyeong Lee, Yong-Seok Choi, Junho H. Lee

**Affiliations:** 1 Department of Biotechnology, Chonnam National University, Gwangju 61186, Republic of Korea; dltlstn39@gmail.com (S.L.); yeomself2355@gmail.com (S.E.); dbswlgnjs128@gmail.com (J.Y.); 2 Organic Agriculture Division, National Institute of Agricultural Sciences, Wanju 55365, Republic of Korea; leeja6311@korea.kr; 3 Bioenvironmental Division, Chungnam Agricultural Research and Extension Services, Yesan 32418, Republic of Korea

**Keywords:** 2,4-di-*tert*-butylphenol (DTBP), odorant receptor, insect control, odorant receptor coreceptor (Orco), two-electrode voltage clamp (TEVC), insect behavior

## Abstract

Odorant molecules interact with odorant receptors (ORs) lining the pores on the surface of the sensilla on an insect’s antennae and maxillary palps. This interaction triggers an electrical signal that is transmitted to the insect’s nervous system, thereby influencing its behavior. Orco, an OR coreceptor, is crucial for olfactory transduction, as it possesses a conserved sequence across the insect lineage. In this study, we focused on 2,4-di-*tert*-butylphenol (DTBP), a single substance present in acetic acid bacteria culture media. We applied DTBP to oocytes expressing various *Drosophila melanogaster* odor receptors and performed electrophysiology experiments. After confirming the activation of DTBP on the receptor, the binding site was confirmed through point mutations. Our findings confirmed that DTBP interacts with the insect Orco subunit. The 2-heptanone, octanol, and 2-hexanol were not activated for the Orco homomeric channel, but DTBP was activated, and the EC_50_ value was 13.4 ± 3.0 μM. Point mutations were performed and among them, when the W146 residue changed to alanine, the E_max_ value was changed from 1.0 ± 0 in the wild type to 0.0 ± 0 in the mutant type, and all activity was decreased. Specifically, DTBP interacted with the W146 residue of the Orco subunit, and the activation manner was concentration-dependent and voltage-independent. This molecular-level analysis provides the basis for novel strategies to minimize pest damage. DTBP, with its specific binding to the Orco subunit, shows promise as a potential pest controller that can exclusively target insects.

## 1. Introduction

**Figure 1:**
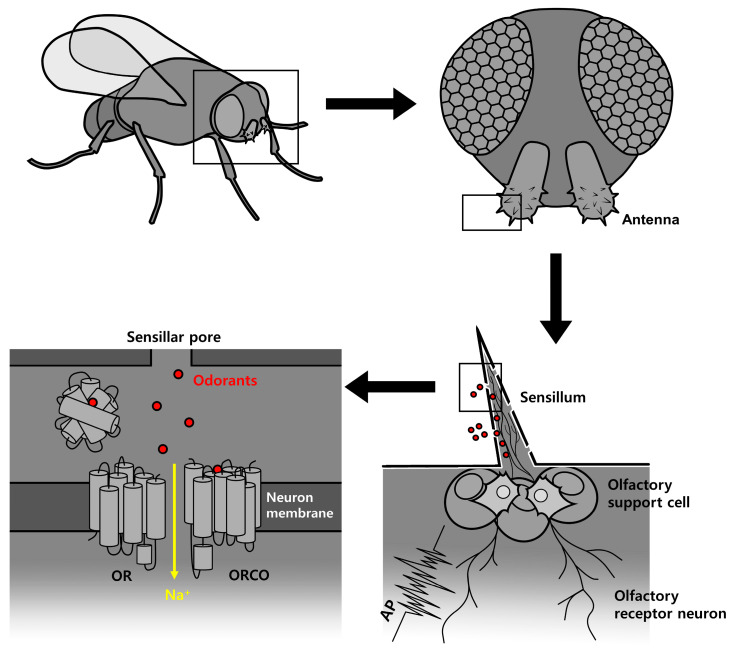
Schematic diagram of the roles and signal transmission of insect odorant receptors (ORs). The insect olfactory sensilla found on the antennae and maxillary palps show dendrites, and ORs are expressed on the dendritic membranes. Upon the binding of odorants to specific ORs, cations flow through the non-selective cation pore. The influx of cations triggers an action potential (AP), which is transmitted along the OR or neuron through the axon to the central nervous system.

The insect olfactory system can be categorized into two major classes, ionotropic receptors (IRs) and odorant receptors (Ors). Among them, Ors specifically detect volatile chemical signals, whereas IRs are related to ionotropic glutamate receptors [1]. Both types are heteromeric ligand-gated cation channels composed of coreceptor proteins and odor-specific receptor proteins. This olfactory system allows insects to recognize and differentiate a wide variety of chemicals present in their environment [2]. Some odorants can act on more than one receptor and the multiple olfactory receptor subunits clustered in the genome are coexpressed [3]. This is the reason why they respond to a broad spectrum of odorants. Figure 1 shows the activation mechanisms of the ORs found in the insect’s antennae, the insect’s maxillary palps, and the larvae’s antennal lobe. Odor molecules activate ORs located within the sensillum pores on the insect’s antennae and maxillary palps, thus generating nerve impulses [4]. These nerve impulses are transmitted to the olfactory sensory neurons (OSNs) through the aqueous medium within the nerve fibers and are then interpreted by the nervous system [5,6,7]. The olfactory system plays a crucial role in determining insect behaviors, such as host plant preference, oviposition site selection, mate choice, and danger avoidance, all of which directly impact their survival [8]. The sensitive and wide-ranging olfactory system is active in detecting and differentiating large numbers of low-mass molecules and most organic compounds. The olfactory repertoire typically includes aliphatic, aromatic molecules, carbon skeletons, and a variety of functional groups (aldehydes, esters, ketones, and alcohols) [9].

ORs are divided into two main subunits: a common subunit known as Orco and another subunit responsible for odorant specificity known as OrX. Orco plays a key role in influencing the composition of ion pores, whereas OrX contributes to the specificity of odor detection [10]. Orco, an OR coreceptor, is widely expressed in OSNs and has a highly conserved sequence across different insect lineages, making it an essential element for olfactory transduction [2,11]. Orco itself is not activated by odorants but responds to agonists [12]. Conversely, OrX is an odorant-binding subunit capable of detecting volatile molecules when present in liposomes. In insect-specific ORs, the OrX subunit exhibits only an approximately 20% similarity both within and across species, which underlines the specificity of the insect olfactory system. Notably, the OrX protein cannot form a homomeric structure without the presence of the Orco protein, and mutations in the OrX subunit can alter their specificity to a particular odorant [2]. Contrariwise, the Orco protein can spontaneously form homomeric cation channels even in the absence of the OrX protein and can be activated by agonists [2]. Therefore, studies exploring how insects distinguish similar odors have revealed a wide variety of behavioral reactions, even in response to pyrazines with similar structures [13].

Insect ORs lack a homology with G-protein coupling with vertebrate chemosensory receptors. The structural dissimilarity between insect ORs and those of humans and other mammals confers high specificity in targeting the Orco subunit for pest control. Given that the agonist binding site of Orco is structurally conserved, both agonists and antagonists targeting the obligatory subunit Orco can be used for pest control across various insect species [14]. Therefore, the conserved structure of Orco makes it a suitable target for a new generation of controllers to combat a wide range of pests.

Pests have a direct impact on both human health and the environment due to their ability to decimate crops and carry diseases. Every year, millions of people become ill from various diseases carried by insects [15]. Additionally, pests can cause serious damage to crops and disrupt the agroeconomic systems of societies. The impacts of insects as vectors of diseases are a major health concern, as exemplified by the wide variety of diseases passed on to humans and other animals through hematophagous arthropods, such as mosquitoes, ticks, and fleas. In response, insecticides have been proposed as a promising solution to control these diseases [16]. However, several studies have demonstrated that the continuous use of typical insecticides leads to the evolution of resistance in pests and insecticide resistance management (IRM) is important for effective pest management [17,18].

To effectively manage pests, the development of a new generation of pest control agents should consider key factors such as affordability, toxicology, efficacy, environmental impact, and resistance [19]. Additionally, it is crucial to avoid the spread of toxic agents through the food chain, which may impact animals and plants beyond the target pests [20]. Thus, exposure levels and potential toxicity must be carefully assessed when developing new control agents against insect pests. Early synthetic organic insecticides have raised environmental and toxicological concerns, leading to their limited use. Therefore, studies should focus on developing new control agents against insect pests that are efficient, stable, and capable of delaying the development of resistance. Particularly, such research should explore molecular-level aspects, including biochemical transformation, potency, and residuality [21].

The compound 2,4-di-*tert*-butylphenol (DTBP) is known to exert various biological effects because of its antifungal, antibiotic, antibacterial, and antioxidant activities, and it is also a common secondary metabolite produced by various groups of organisms [22,23,24,25]. An experimental biocontrol study employing a supplemented fraction of DTBP effectively impeded the proliferation of fungal species on wheat grains. DTBP exhibits potent contact toxicity and repellency against the red flour beetle (*Tribolium castaneum*) and the cigarette beetle (*Lasioderma serricorne*) [26], and it binds to odorant-binding proteins in the antennae of the female scarab beetle (*Holotrichia oblita*) [27]. Furthermore, DTBP elicited a dose-dependent egg-laying preference in gravid mosquitoes [28]. Consequently, it was posited that bacterial symbionts associated with gravid mosquitoes may be transferred to aquatic habitats during egg laying, and together with their volatiles act as ovary-position cues indicating the suitability of active breeding sites to conspecific females [28]. DTBP is believed to play an important role in stimulating the odorant sensing system of insects; thus, studies that enhance both the understanding of the olfactory receptors involved in this cellular signaling process and the mechanism of action are necessary.

In this study, DTBP, a substance found in the culture medium of acetic acid bacteria, was selected due to its insecticidal property against various pests and was applied to oocytes expressing various ORs. Electrophysiology experiments were conducted using a two-electrode voltage clamp (TEVC). Additionally, heteromeric and homomeric ORs from *D. melanogaster* were expressed in *Xenopus laevis* oocytes, after which they were exposed to known odorants that activate each receptor [29,30,31].

## 2. Results

### 2.1. Results of Applying the Odorants and DTBP to Various ORs

**Figure 2:**
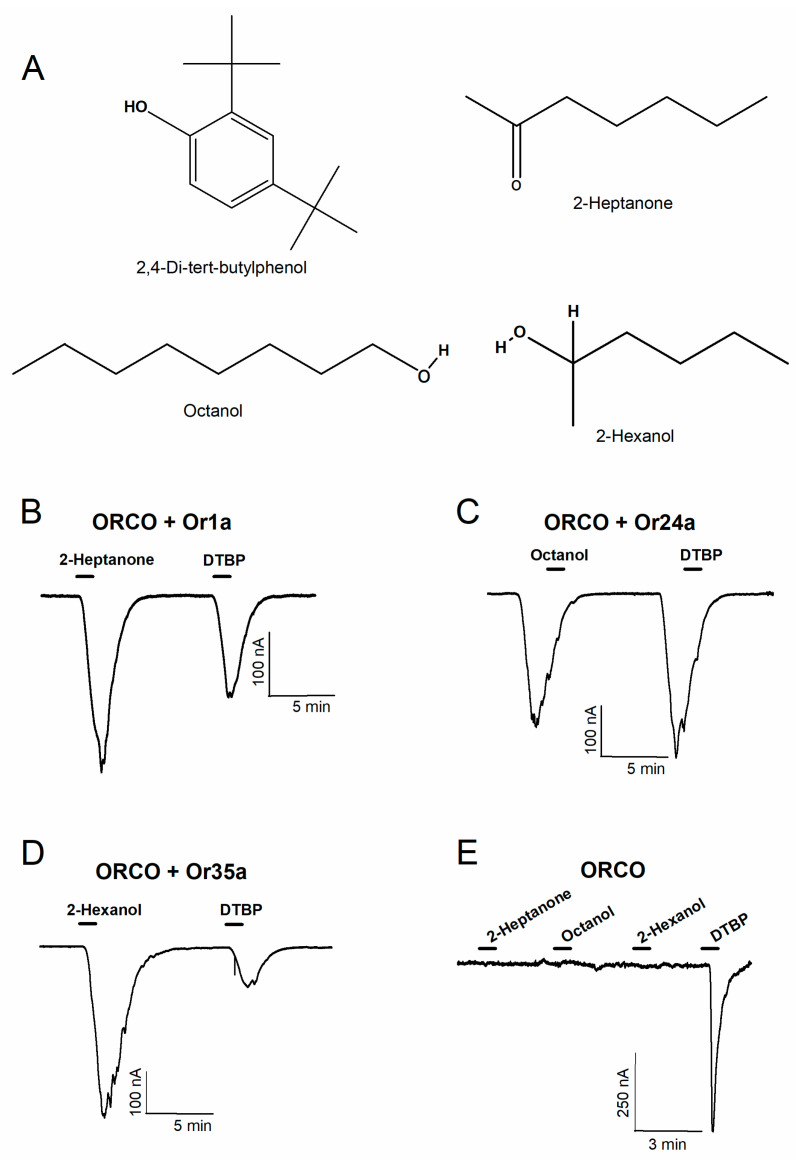
Confirmation of 2,4-di-tert-butylphenol (DTBP) interaction with various odorant receptors in *Drosophila*. (**A**) The chemical structure of DTBP, 2-heptanone, octanol, and 2-hexanol was used to confirm the interaction. (**B**) Confirmation of the interaction between 2-heptanone and DTBP, an odorant for the *D. melanogaster* Orco + Or1a receptor. The concentration of 2-Heptanone was 0.3 μM and DTBP was 30 μM. (**C**) Confirmation of the interaction between octanol, an odorant, and DTBP for the *D. melanogaster* Orco + Or24a receptor. The concentration of Octanol was 0.3 μM and DTBP was 30 μM. (**D**) Confirmation of the interaction between DTBP and 2-hexanol, an odorant for the *D. melanogaster* Orco + Or35a receptor. The concentration of 2-Hexanol was 10 μM and DTBP was 10 μM. (**E**) Confirmation of the interaction between each odorant and DTBP for the *D. melanogaster* Orco homomeric receptor. All experiments were performed at room temperature using a two-electrode voltage clamp, and the holding potential was −80 mV. (**E**) All chemicals were applied at a 100 μM concentration (n = 6–8, obtained from four different frogs).

DTBP isolated from acetic acid bacteria cultures was applied to various D. melanogaster heteromeric receptors and the Orco homomeric receptor. Figure 2A displays the chemical structure of DTBP and other odorants used in the study. Figure 2B–D present the results of the confirmation of receptor expression by applying representative odorants and DTBP to various heteromeric receptors, showing that DTBP binds to all three receptors. Figure 2E shows the results of applying each odorant and DTBP to the Orco homomeric receptor, indicating that DTBP primarily interacts with the Orco subunit rather than with OrX, the odorant-specific subunit of the OR.

**Figure 3:**
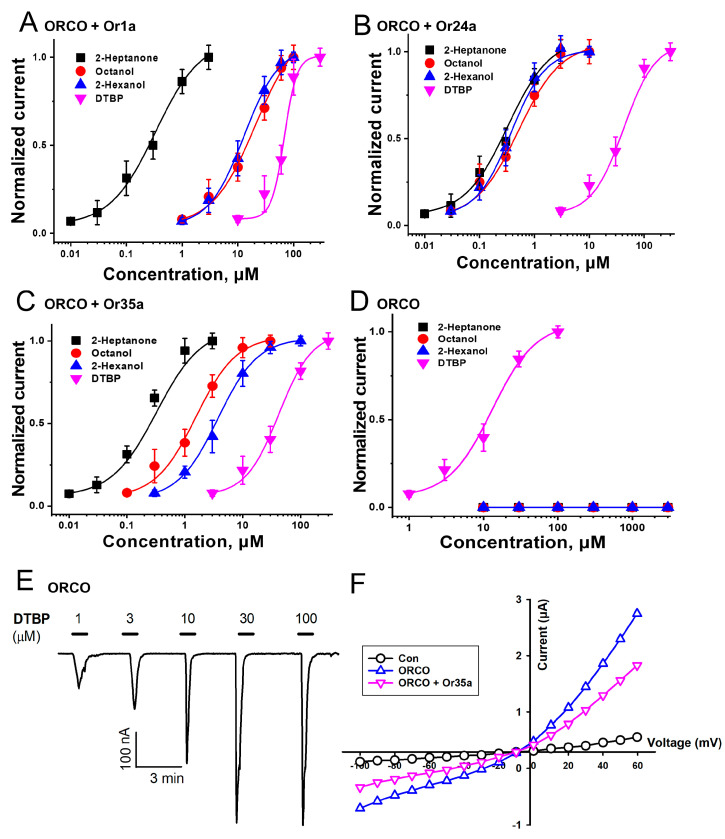
Dose–response curves and activation mechanism of each odorant for various odorant receptors (ORs) in *Drosophila melanogaster*. (**A**–**D**) The graph displays the dose–response curves for each OR, indicating the name of the receptor at the top of the plot. The curves represent the analysis of the inward current obtained through the two-electrode voltage clamp. All applied odorants and 2,4-di-*tert*-butylphenol (DTBP) were applied at a 100 μM concentration. The analysis was fitted using the Hill equation. (**E**) Concentration-dependent activity of DTBP against the Orco homomeric receptor. The applied concentrations are indicated at the top of the inward current. The experiments were performed at room temperature using a two-electrode voltage clamp, and the holding potential was −80 mV. (**F**) Results of applying voltage fluctuations to Orco + Or35a heterodimeric receptor and Orco homomeric receptor to confirm voltage-dependent relationships. The procedure involved the ramp protocol of the two-electrode voltage clamp, with the applied voltage ranging from −100 to +60 mV and applied currents were 30 μM (n = 6–8, obtained from four different frogs).

To verify the pharmacological indicators for the activity of odorants and DTBP on each receptor, current measurements were conducted using representative odorants and DTBP, and the results were normalized and presented as a dose–response curve (Figure 3). Figure 3A–C illustrate the normalized activation of DTBP and other odorants for each heteromeric receptor. In Figure 3D, 2-heptanone, octanol, and 2-hexanol were not activated for the Orco homomeric channel, but DTBP was activated, and the EC_50_ value was 13.4 ± 3.0 μM. These results confirm that DTBP has a higher affinity for the Orco subunit.

### 2.2. Confirmation of the DTBP Activation Mechanism on Heteromeric and Homomeric Receptors

As illustrated in Figure 3E, the activation mechanism of DTBP binding to the Orco homomeric receptor was investigated by applying DTBP at different concentrations. The results confirmed that DTBP exhibits a concentration-dependent effect on the Orco homomeric receptor. Table 1 shows the DTBP activation according to each type of receptor, and the E_max_ (maximal effective-response at high concentration), EC_50_ (half-maximal effective-response concentration), and n_H_ (coefficient of interaction) values are shown. Additionally, our study sought to determine whether the binding activity of DTBP to each receptor is affected by voltage fluctuations. By applying different voltages ranging from −100 to +60 mV, our results confirmed that the activity of DTBP varies in a voltage-independent manner and is not affected by changes in the current slope according to voltage fluctuations (Figure 3F).

### 2.3. Confirmation of Interaction between DTBP and Orco Homomeric Receptor

**Figure 4:**
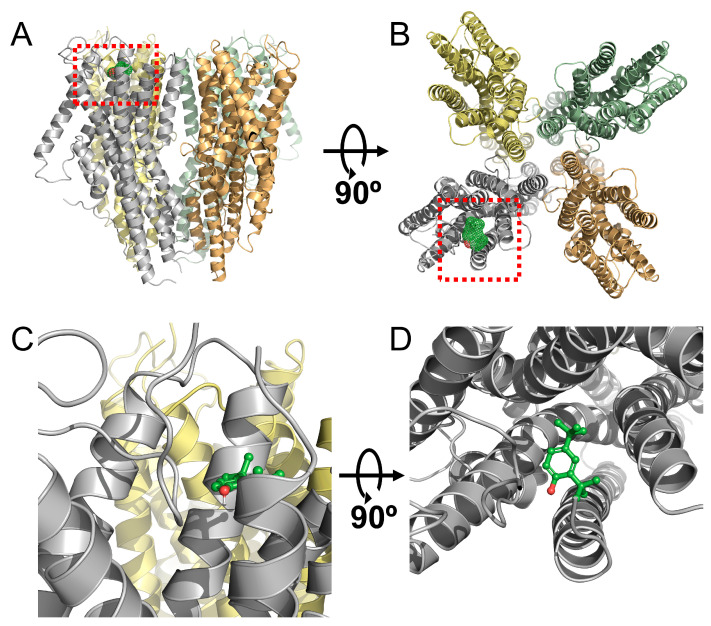
Confirmation of the interaction between 2,4-di-*tert*-butylphenol (DTBP) and the three-dimensional (3D) protein structure of the Orco homomeric receptor through docking modeling. (**A**) Interaction between the Orco homomeric receptor and DTBP viewed from the front. (**B**) Interaction between the Orco homomer receptor and DTBP viewed from the top. This view is rotated 90° from the view in (**A**). (**C**) Confirmation of the interaction pocket site in the Orco subunit. (**D**) This view is rotated 90° from the view in (**C**). To visualize the interaction, the Orco subunit was represented as a tertiary structure of a protein, and DTBP was represented as a ball-and-stick structure.

Figure 4 presents a 3D protein structure that illustrates the interaction between the Orco homomeric receptor and DTBP. The Orco subunit autonomously forms a homotetrameric receptor even in the absence of the OrΧ subunit. To verify the presence of the DTBP binding site within the Orco subunit, a 3D protein structure was constructed and subjected to docking simulation to identify the lowest energy binding site. The figure illustrates the interaction between DTBP and the Orco homotetrameric receptor, taking into consideration the relationship between the Orco subunit and DTBP. The binding site of DTBP exists in the binding pocket in the Orco subunit.

### 2.4. Identification of the Binding Site in the Orco Homomeric Receptor of DTBP through Point Mutations

**Figure 5:**
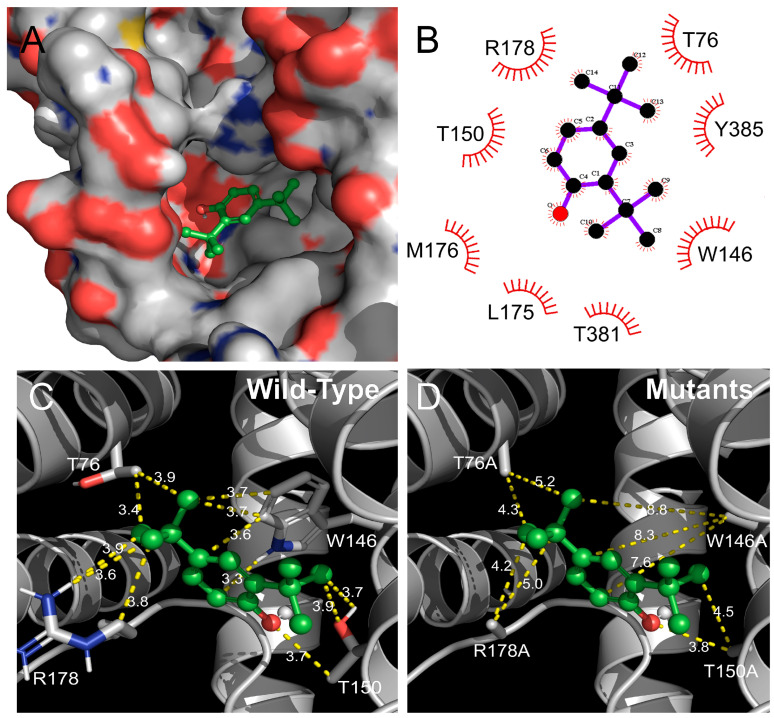
Interaction of 2,4-di-*tert*-butylphenol (DTBP) with a wild-type Orco subunit and confirmation of the interaction distance change on mutant-type Orco subunit. (**A**) Illustration of the interaction of DTBP with a wild-type Orco protein. (**B**) Representation of the chemical structure of the interaction between the Orco subunit residue and DTBP. (**C**) Visualization of the interaction between DTBP and the wild-type Orco subunit, showing the interaction distances and residues involved. (**D**) Visualization of the interaction between DTBP and the mutant-type Orco subunit, indicating the changes in interaction distances and residues. The figure displays the interaction distances and residues of the Orco subunit and DTBP.

Figure 5A depicts the interaction of DTBP with the Orco homomeric receptor at the molecular level. In the figure, the spheres represent Orco subunits, whereas the ball-and-stick models represent DTBP. Figure 5B presents a schematic diagram illustrating the subunit residues that interact with DTBP. Figure 5C,D display the interaction distances between the Orco subunit and DTBP, obtained via the AutoDock 4.0 program. Figure 5C shows the interaction between the wild-type Orco protein and DTBP. Figure 5D shows the interaction distance of the mutant-type Orco protein and DTBP. In the wild-type, DTBP interacts with four residues: (1) T76, distances = 3.4 and 3.9 Å; (2) R178, 3.9, 3.6, and 3.8 Å; (3) T150, 3.7, 3.9, and 3.7 Å; and (4) W146, 3.3, 3.6, 3.7, and 3.7 Å. In the mutant-type, the interaction distance of DTBP was affected by the following four residues: (1) T76, distances = 4.3 and 5.2 Å; (2) R178, 4.2 and 5.0 Å; (3) T150, 3.8 and 4.5 Å; and (4) W146, 7.6, 8.3, and 8.8 Å. The molecular interactions and each distance are shown in the figures.

### 2.5. Confirmation of Changes in DTBP Activity in Mutant-Type Receptor

**Figure 6:**
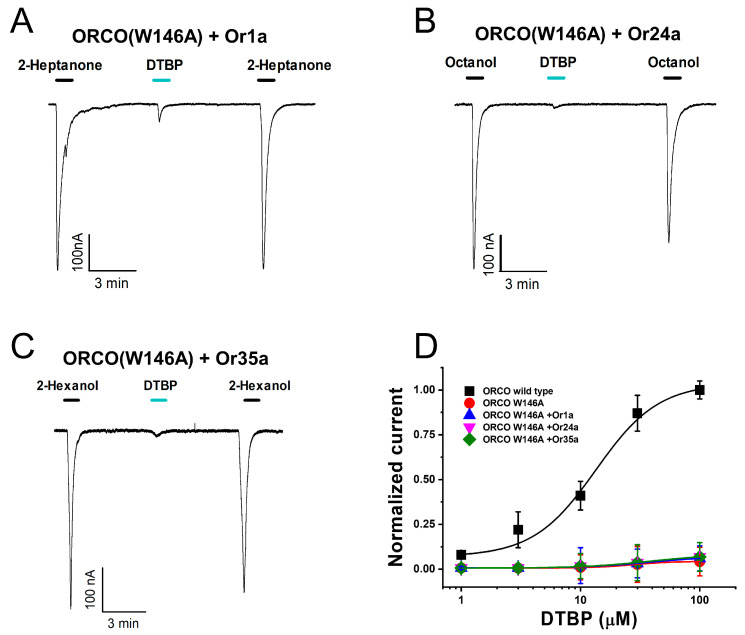
Confirmation of induced inward current by each odorant and 2,4-di-*tert*-butylphenol (DTBP) according to the mutant-type (W146A) Orco subunit + wild-type OrX subunit in each *Drosophila melanogaster* odorant receptor. (**A**–**C**) Confirmation of inward current by odorant and DTBP according to each receptor. The name of each receptor is displayed at the top of the graph. The experiments were conducted using the ramp protocol of the two-electrode voltage clamp, and the holding potential was −80 mV (n = 6–8, obtained from four different frogs). (**D**) Dose–response curve for DTBP in the Orco wild-type and mutant-type. DTBP was applied at a 100 μM concentration. The analysis was fitted using the Hill equation.

Through point mutations, a tryptophan (W) residue was replaced with alanine (A) in the potent binding site of DTBP, after which these changes were verified at the molecular level. For each receptor, wild-type OrX (Or1a, Or24a, and Or35a) and mutant-type Orco were co-injected into oocytes to induce coexpression, followed by DTBP application. Figure 6A–C illustrate the results of applying the odorants and DTBP. Our findings confirmed that only the inward current induced by DTBP decreased, whereas the current induced by the odorant, which was previously applied to the wild-type receptor, remained the same. This finding confirms that DTBP binds to the W146 residue of the Orco subunit. Figure 6D represents the result of normalizing the current activity using DTBP. In the Orco W146A mutant-type, the E_max_ value changed from the wild-type’s 1.0 ± 0.0 to 0.0 ± 0.0. These results further confirmed that the binding site of DTBP is the W146 residue of the Orco subunit, and the OrX subunit does not have a binding site and is not affected by DTBP.

## 3. Discussion

In this study, DTBP extracted from acetic acid bacteria cultures was tested on various receptors of *D. melanogaster*’s ORs. Our findings confirmed that DTBP acts as an agonist specifically for the Orco subunit. DTBP exhibited activity on all three receptors (Orco + Or1a, Orco + Or24a, and Orco + Or35a) and the Orco homomeric receptor. However, the OrX odorants (2-heptanone, octanol, and 2-hexanol) did not activate the Orco homomeric receptor, indicating that DTBP’s activity is specific to the Orco subunit and does not affect the OrX subunit. As DTBP acts as an agonist for the Orco homomeric receptor regardless of the presence of the OrX subunit, both the Orco + OrX heteromeric and the Orco homomeric receptors share the same (concentration-dependent) activation mechanism. Additionally, our findings indicated that this activation is voltage-independent, meaning that ion entry and exit are not influenced by changes in voltage but rather depend on the receptor type and the applied DTBP concentration. Through protein-ligand docking modeling, the W146 residue was identified as the specific binding site of DTBP in the Orco subunit. Point mutation experiments, where the W146 residue was replaced with alanine, decreased the DTBP’s activity. Notably, the substitution of this residue did not affect the activity of other OrX odorants, confirming that the W146 site is specific to DTBP binding. These findings suggest that DTBP may elicit attractive effects in insects. Furthermore, the acetic acid bacteria cultures contained many single substances that could potentially serve as insect control candidates. Our study successfully confirmed that DTBP acts as an Orco subunit-specific agonist, which opens up possibilities for discovering other attractants that can target the Orco subunit without affecting species-specific OrX receptors.

Orco, a highly conserved coreceptor, exhibits a conserved sequence across lineages. Through topology and entropy value analysis, researchers confirmed the high conservation of the C-terminal region of the insect Orco protein [2]. The Orco genes have been preserved for an impressive 250 million years, supporting their primary functions in extracellular signal transduction to the central nervous system and heterodimerization with various OrΧ proteins [32]. However, throughout its evolutionary history, the C-terminal region has exhibited few variations in response to species-specific OrΧ proteins. In contrast, the N-terminal region shows the potential for continuous evolution in accordance with lineage-specific OrΧ proteins. The N-terminal region of the Orco protein acts as a template that evolves over time to accommodate the specific OrΧ protein to be assembled, and therefore different insect species can exhibit different responses to the same odorant [32]. Research has shown that the Orco subunit can be replaced with the OrΧ subunit [33]. Insect odorant-specific responses are primarily influenced by the OrΧ subunit, whereas the Orco proteins, which are conserved across insect lineages [2], form the basic scaffold. The OrΧ subunit, which has evolved based on the habitat of insects, then heterodimerizes with the Orco subunit. When mapped to the structure of Orco, the more conserved residues line up primarily inside the pore and form clusters within the anchor domain. The use of ConSurf protein evolutionary data confirmed that the pore part and inter-subunit contact residue of the Orco protein were highly conserved. [34,35,36]. These conserved regions are positioned closer to the C-terminal region pore in the 3D structure of the Orco homotetramer. These findings suggest that the preservation of these conserved sequences across insect lineages is crucial for several basic functions, such as signal transduction, ion flux, and heterodimerization with various OrΧ proteins [32].

The increasing risk of disease transmission by pests, such as tsetse flies in Sub-Saharan Africa, poses a serious threat to humans and livestock. Climate change is expected to expand the disease’s reach to new areas with relatively greater human and livestock populations [37]. To control these pests, an understanding of the neural coding and molecular bases of the olfaction system is emerging. This is based on insects’ use of volatile organic compounds (VOCs) for communication within the species and for locating various resources [38]. The sensitivity of the olfactory system is essential to the survival of the insect. Mosquitoes are highly dependent on the olfactory system to seek plant sources, hosts for blood meal, and breeding sites, and after feeding on blood the transcript levels for specific genes, including odorant binding receptors, were changed [39,40]. Drosophila melanogaster larvae were strongly attracted to highly diluted ethyl acetate in a closed laboratory, but the addition of the airborne Orco antagonist OX1w to the laboratory abolished larval chemotaxis toward ethyl acetate [30]. Based on these results, the regulation of odorant receptors can change the olfactory behavior of insects. Therefore, using chemoattractants that activate insect olfactory systems can be an efficient approach. Chemoattractants for insects can be applied in diverse ways, such as using them to treat food waste to attract fruit fly larvae [41].

Controlling pests through the manipulation of the olfactory behavior of insects is considered a promising strategy. The odorant-specific subunit OrX has been targeted for insect control in some instances. However, it is crucial to note that this subunit possesses species-specific characteristics. Particularly, the expression of different proteins by the OrX subunit depends on the insect species, resulting in specific responses to various odorants, attractants, or repellents. Consequently, targeting this subunit poses challenges and limitations. In contrast, the Orco subunit is present in all olfactory systems and stands apart from traditional ORs due to its highly conserved characteristics across different species. This suggests that the Orco subunit plays a crucial role in various insect species.

Collectively, our findings demonstrated that the Orco subunit is not activated by odorants, but that DTBP acts as an agonist. These findings suggest that targeting an agonist for Orco can modulate olfactory behavior, thus allowing for the discovery of substances that can activate or inhibit the Orco subunits as controllers beyond the limits of a particular species. Even when there is a substantial amount of OR-specific volatile molecules in the external environment, the activation or inhibition of the Orco subunit can alter odorant-induced olfactory behavior by binding to the OrX subunit [30]. The similar pattern of agonist and antagonist sensitivity exhibited by Orco subunits from a variety of other species suggests that the binding site of Orco has a highly conserved structure. Since OR has no similarity with human or other mammalian receptors, it can work in an insect-specific attractive or aversive manner [1,29]. The mechanism of action of DEET, a widely used repellent around the world, has been found to be the inhibition of the highly conserved Orco subunit [42]. Therefore, leveraging the expression of ORs in insects, which varies depending on the species and their environment, can be a viable strategy for protecting crops and other animals from harmful insecticides [43]. Recent studies have revealed that DEET also affects the insects’ gustatory receptor (GR) [44]. We focused on the olfactory receptors of insects, but as the importance of the GR is also emerging, their synergy can also be studied.

## 4. Materials and Methods

### 4.1. Materials

2,4-Di-tert-butylphenol (DTBP) from acetic acid bacteria cultures was selected as a representative chemoattractant in this study due to its known insect attractant effect. Single chemicals were analyzed to identify and choose the microbial metabolites with the most significant attracting effect. DTBP, 2-heptanone, octanol, 2-hexanol, and all other reagents used in this study were purchased from Sigma-Aldrich (St. Louis, MO, USA). For the TEVC experiment, all reagents were appropriately diluted using DMSO, with the final DMSO concentration not exceeding 0.01%.

### 4.2. In Vitro Transcription

*D. melanogaster* OrX subunit (Or1a, Or24a, and Or35a) (FlyBase ID: FBgn0029521, FBgn0026394 and FBgn0028946) and Orco (odorant receptor coreceptor) (FlyBase ID: FBgn0037324) receptor cDNA were obtained from gene synthesis using Cosmogenetech (14, Seongsui-ro10-gil, Seongdong-gu, Seoul, Republic of Korea) and the cDNA was synthesized using p-GEM-HE vector. The DNA transformation experiment was performed with DH5α competent cell. The transformed cDNA was harvested using miniprep experiment and their sequence was confirmed. For cRNA synthesis, *D. melanogaster* Or1a, Or24a, Or35a, and Orco cDNA were linearized with appropriate restriction enzymes, after which electrophoresis was conducted to confirm that they were properly cleaved. In vitro transcription was performed using T7 RNA polymerase (mMESSAGE mMACHINE T7 Transcription Kit; Thermo Fisher Scientific, Waltham, MA, USA). The final mRNA pellet was suspended in DEPC-treated water and stored at −80 °C until use.

### 4.3. Xenopus Oocyte Preparation and mRNA Injection

Two-electrode voltage clamp (TEVC) experiments were performed using *Xenopus* oocytes. *Xenopus laevis* frogs were obtained from the Korean Xenopus Resource Center for Research (KXRCR000001, Chuncheon-si, Gangwon-do, Rupublic of Korea). The *Xenopus* oocyte preparation and maintenance protocols were the same as those described in a previous study [45], and were conducted in accordance with the high standards of Chonnam National University’s institutional guidelines (CNU IACUC-YB-2016-07, July 2016). A nanoliter injector (Drummond Scientific, Vernon Hills, IL, USA) was used to inject a total of 50 nl cRNA into each oocyte. The injected oocytes were then incubated in a shaking incubator at 18 °C to ensure proper expression prior to conducting the experiments.

### 4.4. Electrophysiological Recording Using a Two-Electrode Voltage Clamp

The interaction between the odorant receptors, odorants, and agonists was analyzed at the molecular level using the oocytes expressing the specific receptors. Odorants-induced inward currents were recorded using a two-electrode voltage clamp setup (OC-725C; Warner Instruments, Hamden, CT, USA) and Digidata (1550S; Molecular Devices, Sunnyvale, CA, USA). The membrane holding potential of the oocyte was artificially fixed at −80 mV. Each agent stock was dissolved in ND96 buffer (58.44 mM NaCl, 2 mM KCl, 1 mM MgCl_2_, 1.8 mM CaCl_2_, 5 mM HEPES, pH 7.4) at the appropriate concentration and perfused at a 2 mL/min rate. The induced inward current was converted into digital data, and current–voltage (I–V) relationships were acquired and analyzed using the pClamp10 software (Axon Instruments, Union City, CA, USA). All data were acquired using the Clampex 10 software at a sampling rate of 2 kHz.

For voltage relationship experiments to confirm the relationship between induced internal currents and voltage, a ramp protocol was used to vary the voltage from −80 mV to +60 mV.

### 4.5. Site-Directed Mutagenesis to Verify Interaction Sites

After identifying potential binding sites through 3D protein docking modeling, molecular verification was performed using site-directed mutagenesis. Point mutations of the insect odorant receptor were introduced through polymerase chain reaction (PCR). Primers were designed appropriately to change the amino acid residues to be changed, and the products were obtained through PCR. The PCR product was transformed into XL-1-Blue super competent cells, after which miniprep was conducted. The changes in the pDNA sequence were confirmed by Gentech Inc. (Seoul, Seondong-gu, Rupublic of Korea). To confirm the binding site of DTBP at the molecular level, the Orco subunit of each mutant type was co-injected with wild-type OrX and induce expression. The affinity change of DTBP was confirmed with TEVC.

### 4.6. 3D Protein Structure Composition and Molecular Interaction Confirmation

The protein structures for the molecular docking study of *D. melanogaster*’s Cryo-EM structure of Orco (ID code 6C70, 3.50 Å resolution) were obtained from the Protein Data Bank. The 3D model of 2,4-Di-tert-butylphenol (DTBP) was obtained from the PubChem (PubChem CID: 7311) database. Molecular docking studies were conducted using Autodock Tools (version 4.2.6, La Jolla, CA, USA) from The Scripps Research Institute, considering intermolecular energy, inhibition constant, crystal structures, and energy minimization. DTBP was docked using the basic settings of the Autodock program. The protein complex of DTBP and the Orco homotetrameric receptor was further analyzed using Ligplot (version 4.5.3, EMBL-EBI, Hinxton, Cambridgeshire, UK) and Pymol (version 1.8.4.2, Schrödinger, New York, NY, USA).

### 4.7. Data Statistics and Analysis

The experiments were performed more than three times for scientific validation, and the data are presented as the mean ± standard error of the mean (SEM). All data collected from the two-electrode voltage clamp experiments were analyzed using the SigmaPlot 10.0 software. The dose–response curve data were visualized using OriginPro 9.0 software (Origin, MA, USA) and the I–V relationship was fitted using the Hill equation:

y = V_min_ + (V_max_ + V_min_) × [x]^n^/([EC_50_]^n^ + [x]^n^)

where V_max_ and V_min_ are the maximum and minimum current, x is the concentration of the applied ligand, and n is the coefficient of interaction. EC_50_ is the half-maximal effective-response concentration and E_max_ is the maximal effective-response at high concentration when all the receptors are occupied by odorants and DTBP. *p*-values < 0.05 were considered statistically significant.

## Figures and Tables

**Table 1:** Effects of DTBP on various types of insect odorant receptors.

Type	E_max_	EC_50_	n_H_
ORCO + Or1a	1.1 ± 0.1	104.4 ± 27.7	1.1 ± 0.2
ORCO + Or24a	1.0 ± 0.1	51.0 ± 8.2	1.2 ± 0.3
ORCO + Or35a	1.0 ± 0.0	38.1 ± 3.8	1.3 ± 0.2
ORCO	1.0 ± 0.0	13.4 ± 3.0	1.7 ± 0.7

## Data Availability

Raw sequences obtained using the Nucleotide database have been made available through the NCBI Sequence Library. The datasets generated during and/or analyzed during the current study are available from the corresponding author on reasonable request.
